# Immunophenotype based on inflammatory cells, PD-1/PD-L1 signalling pathway and M2 macrophages predicts survival in gastric cancer

**DOI:** 10.1038/s41416-020-01053-7

**Published:** 2020-09-18

**Authors:** Anna Junttila, Olli Helminen, Juha P. Väyrynen, Maarit Ahtiainen, Istvan Kenessey, Sirpa Jalkanen, Jukka-Pekka Mecklin, Ilmo Kellokumpu, Teijo Kuopio, Jan Böhm, Johanna Mrena

**Affiliations:** 1 Department of Surgery, Central Finland Central Hospital, Jyväskylä, Finland; 2 Cancer and Translational Medicine Research Unit, Medical Research Center Oulu, University of Oulu, and Oulu University Hospital, Oulu, Finland; 3 Department of Medical Oncology, Dana-Farber Cancer Institute and Harvard Medical School, Boston, MA USA; 4 Department of Pathology, Brigham and Women’s Hospital, Boston, MA USA; 5 Department of Education and Research, Central Finland Health Care District, Jyväskylä, Finland; 6 MediCity Research Laboratory and Institute of Biomedicine, University of Turku, Turku, Finland; 7 Department of Education and Research, Central Finland Central Hospital and Sport and Health Sciences, University of Jyväskylä, Jyväskylä, Finland; 8 Department of Pathology, Central Finland Central Hospital, Jyväskylä, Finland; 9 Department of Biological and Environmental Science, University of Jyväskylä, Jyväskylä, Finland

**Keywords:** Immunosurveillance, Molecular medicine

## Abstract

**Background:**

Immune response against cancer has prognostic impact but its role in gastric cancer is poorly known. The aim of the study was to assess the prognostic significance of immune cell score (CD3+, CD8+), tumour immune escape (PD-L1, PD-1) and immune tolerance (Clever-1).

**Methods:**

After exclusion of Epstein-Barr virus positive (*n* = 4) and microsatellite instable (*n* = 6) tumours, the study included 122 patients with GC undergoing D2 gastrectomy. CD3+ and CD8+ based ICS, PD-L1, PD-1 and Clever-1 expressions were evaluated. Differences in survival were examined using Cox regression adjusted for confounders. The primary outcome was 5-year survival.

**Results:**

The 5-year overall survival rate was 43.4%. High ICS was associated with improved overall survival (adjusted HR 0.48 (95% CI 0.26–0.87)) compared to low ICS. In the high ICS group, patients with PD-L1 expression (5-year survival 69.2 vs. 53.1%, *p* = 0.317), high PD-1 (5-year survival 70.6 vs. 55.3% *p* = 0.312) and high Clever-1 (5-year survival 72.0% vs. 45.5% (*p* = 0.070) had poor prognosis.

**Conclusions:**

High ICS was associated with improved survival. In the high ICS group, patients with high PD-L1, PD-1 and Clever-1 had poor prognosis highlighting the importance of immune escape and immune tolerance in GC.

## Background

Gastric cancer (GC) has the fifth (5.7%) highest incidence of all cancers worldwide and the third (8.2%) highest cancer-related mortality.^1^ Of the several classification systems for gastric cancer, the Laurén classification divides GC into intestinal or diffuse by histological morphology. Together with the TNM (tumour, node and metastasis) system, it is still used in clinical decision-making and prognostic classification.^2,3^ The Cancer Genome Atlas Research Network recently proposed a molecular GC classification into four subtypes: (1) positive for Epstein-Barr virus (EBV), (2) high level of microsatellite instability (MSI), (3) genomically stable and (4) chromosomally unstable, showing that gastric adenocarcinomas comprise diverse molecular backgrounds leading to various clinical courses.^4^

Immunoscore^5^ measures densities of CD3+ and CD8+ lymphocytes at the tumour centre and at the invasive margin.^5,6^ This has been internationally validated in colorectal cancer and its inclusion as a part of TNM-staging (TNMi) has been proposed, based on findings of greater relative prognostic value than TNM staging.^6^ Molecular predictors of survival and therapy response have been intensively researched in GC, but apart from *HER2* testing, molecular tests have not been commonly used in routine clinical practice.^7,8^ Certain studies have shown that a combination of immune cell scoring systems and TNM classification has better prognostic value than TNM staging alone.^9^ However, the optimal method for immune cell scoring is not clear.^10,11^

Immunotherapy has recently emerged as a promising strategy in the treatment of various types cancer.^12^ Programmed cell death ligand 1 (PD-L1) is the ligand for programmed death 1 (PD-1), which is expressed in T cells. Upregulation of PD-L1 occurs in various tumour types, and signalling through this pathway can lead to inhibition of T cell immune response against tumour cells in vitro and in murine models.^13,14^ It has been suggested that an active PD-L1 mechanism promotes immune escape by dumping host cytotoxic immune reaction in gastric cancer patients.^14–17^ High PD-L1 is associated with poor survival in gastro-oesophageal carcinomas,^15–17^ a combination of low immune cell score and high PD-L1 expression is associated with the poorest overall and disease-specific survival rates.^18,19^ As a crucial factor of host immunity modulator, the expression of PD-L1 represents a potential immunotherapy target in gastric cancer.^20^

Immune escape of cancer cells caused by regulatory T cells and immunosuppressive (M2) macrophages has moreover been recognised to contribute to cancer prognosis.^21–23^ Common lymph endothelial and vascular endothelial receptor-1 (Clever-1) is a multifunctional adhesion and scavenger receptor expressed by M2 macrophages.^21^ Similar to high tumour PD-L1 expression, M2 macrophage infiltrate may inhibit T cell response causing increased immune tolerance and poorer prognosis.^17^

The aim of the present study was to assess the prognostic significance of the host immunity elements in GC by measuring immune cell score (CD3+ and CD8+ cells), PD-L1/PD-1 tumour immune escape pathway and immune tolerance mediated by Clever-1 positive M2-like macrophages.

## Methods

### Patients

All patients with histologically confirmed gastric adenocarcinoma (*n* = 132) undergoing D2 gastrectomy in Central Finland Central Hospital during the period 1997–2016 were included in the study. R2 resections were excluded. Clinical data and patient survival data were obtained from patient records. Survival information was confirmed from the Cause of Death Registry maintained by Statistics Finland. The study was duly approved by the hospital district. Use of the samples and patient data were approved by the Ethics Committee and by the National Authority for Medicolegal Affairs (VALVIRA). The preoperative protocol consisted of endoscopy with biopsies and routine thoracoabdominal computed tomography (CT). Endoscopic ultrasound was performed to assess the need for perioperative chemotherapy in patients with endoscopically small, superficial tumours, or when the possibility of less invasive, endoscopic treatments was considered for fragile patients. Positron emission tomography (PET) CT was performed selectively in cases of large tumours, or suspicion of distant metastases or lymph node spread in CT. Accordingly, diagnostic laparoscopy was performed selectively to exclude peritoneal metastases or to supplement inconclusive radiological staging. The exercise tolerance of the patients was tested with stair climbing and their comorbidities were evaluated. From 2010, patients with >T1b tumours amenable to treatment received perioperative chemotherapy according to ESMO guidelines.^24^ Patients were restaged before surgery with CT or PET-CT according to primary fluorodeoxyglucose avidity. The operation was usually performed after a 6-week recovery period by specialised upper gastrointestinal surgeons. The follow-up ended on 5 September 2019. Detailed patient characteristics are presented in Table 1.

**Table 1:** Clinical characteristics according to Lauren classification.

	Lauren classification: diffuse *n* = 70				Lauren classification: intestinal *n* = 48			
Variables	Immune cell score 0	Immune cell score 1–2	Immune cell score 3–4	*p*-value	Immune cell score 0	Immune cell score 1–2	Immune cell score 3–4	*p*-value
Sex				***p* = 0.762**				***p* = 0.379**
Male	*n* = 10	*n* = 13	*n* = 11		*n* = 8	n = 9	*n* = 10	
Female	*n* = 13	*n* = 11	*n* = 12		*n* = 4	n = 5	*n* = 12	
Age				***p* = 0.724**				***p* = 0.751**
<65	*n* = 12	*n* = 9	*n* = 11		*n* = 4	*n* = 3	*n* = 5	
65–75	*n* = 6	*n* = 10	*n* = 9		*n* = 5	*n* = 4	*n* = 7	
>75	*n* = 5	*n* = 5	*n* = 3		*n* = 3	*n* = 7	*n* = 10	
ASA				***p* = 0.062**				***p* = 0.017**
ASA1–2	*n* = 16	*n* = 8	*n* = 12		*n* = 6	*n* = 1	*n* = 3	
ASA3–4	*n* = 7	*n* = 15	*n* = 11		*n* = 6	*n* = 12	n = 19	
Tumour				***p* = 0.020**				***p* = 0.060**
T0	*n* = 0	*n* = 1	*n* = 0		*n* = 0	*n* = 0	*n* = 0	
T1	*n* = 1	*n* = 1	*n* = 8		*n* = 0	*n* = 3	*n* = 7	
T2	*n* = 2	*n* = 6	*n* = 5		*n* = 3	*n* = 7	*n* = 6	
T3	*n* = 15	*n* = 10	*n* = 8		*n* = 9	*n* = 4	*n* = 7	
T4	*n* = 5	*n* = 6	*n* = 2		*n* = 0	*n* = 0	*n* = 2	
Node				***p* = 0.004**				***p* = 0.732**
N0	*n* = 2	*n* = 8	*n* = 14		*n* = 3	*n* = 6	*n* = 7	
N1	*n* = 11	*n* = 13	*n* = 6		*n* = 5	*n* = 7	*n* = 11	
N2	*n* = 9	*n* = 2	*n* = 2		*n* = 2	*n* = 1	*n* = 2	
N3	*n* = 1	*n* = 1	*n* = 1		*n* = 2	*n* = 0	*n* = 2	
Metastasis				***p* = 0.122**				***p* = 0.566**
M0	*n* = 21	*n* = 24	*n* = 23		*n* = 11	n = 14	*n* = 21	
M1	*n* = 2	*n* = 0	*n* = 0		*n* = 1	n = 0	*n* = 1	
Stage				***p* = 0.003**				***p* = 0.368**
I	*n* = 1	*n* = 5	*n* = 12		*n* = 1	*n* = 6	*n* = 7	
II	*n* = 9	*n* = 13	*n* = 7		*n* = 7	*n* = 7	*n* = 8	
III	*n* = 11	*n* = 6	*n* = 4		*n* = 3	*n* = 1	*n* = 6	
IV	*n* = 2	*n* = 0	*n* = 0		*n* = 1	*n* = 0	n = 1	
Tumour location				***p* = 0.533**				***p* = 0.656**
Proximal	*n* = 2	*n* = 4	*n* = 4		*n* = 3	*n* = 4	*n* = 5	
Body	*n* = 9	*n* = 10	*n* = 12		*n* = 4	*n* = 4	*n* = 10	
Distal	*n* = 8	*n* = 7	*n* = 7		*n* = 4	*n* = 6	*n* = 7	
Linitis plastica	*n* = 4	*n* = 3	*n* = 0		*n* = 1	*n* = 0	*n* = 0	
Resection				***p* = 0.150**				***p* = 0.547**
R0	*n* = 20	*n* = 23	*n* = 23		*n* = 12	*n* = 14	*n* = 21	
R1	*n* = 3	*n* = 1	*n* = 0		*n* = 0	*n* = 0	*n* = 1	

### Histopathological examination

The histological diagnosis according to Lauren Classification was confirmed by an expert gastrointestinal pathologist (JB). Tumour stage was determined according to the 7th edition of the UICC/AJCC TNM categories.^3^

### Immunohistochemical analyses

For immunohistochemistry (IHC), formalin-fixed paraffin-embedded tissue sections of 3 µm thickness from the representative tumour tissue block were used. For immune cell score, sections were stained with anti-CD3 (LN10, 1:200; Novocastra) and anti-CD8 (SP16, 1:400; Thermo Scientific) using a LabVision Autostainer 480 (ImmunoVision Technologies Inc.). Antigen retrieval was done with Tris-EDTA buffer at pH 9 in a microwave at 98 degrees Celsius for 15 mins. Samples were incubated with diluted antibodies for 30 mins at room temperature). Staining for PD-1 and PD-L1 was conducted with anti-PD-1 (SP269, 1:50; Spring Bioscience) and anti-PD-L1 (E1L3N, 1:100; Cell Signaling Technology) antibodies, using a BOND-III stainer (Leica Biosystems). Antigen retrieval was done with Tris/EDTA (BOND ER solution 2, pH 9; Leica Biosystems) and samples were incubated with diluted antibodies for 30 mins at room temperature. For staining for Clever-1, the deparaffinised sections were treated with proteinase K (DAKO S3020) for 10 mins at room temperature. Non-specific binding sites were blocked with 2% normal rabbit serum, where after the samples were incubated overnight with rat anti-Clever-1 antibody (2–7) or a class-matched negative control antibody (MEL-14) at +4 °C. For all IHC samples, diaminobenzidine (DAB) was used as a chromogen and haematoxylin as a counterstain. Positive control tissue for CD3, CD8, PD-1, PD-L1 and Clever-1 immunohistochemistry was normal tonsil and for EBV ISH, EBV-positive Hodgkin lymphoma tissue was used.

Mismatch repair (MMR) status was determined by IHC analysis for expressions of MLH1, MSH2, MSH6 and PMS2 as described earlier.^25^ MMR IHC was assessed visually by an experienced researcher (MA) and uncertain cases were confirmed by an experienced pathologist (JB). Absolute absence of nuclear staining was considered MSI. EBV encoded RNA was detected using BOND in situ hybridisation (ISH) EBER probe with BOND Polymer Refine Detection kit (both Leica Microsystems) according to manufacturer’s instructions.

### Scoring

IHC-stained slides were scanned with a NanoZoomer-XR (Hamamatsu Photonics) at ×20 magnification.

**Fig. 1:**
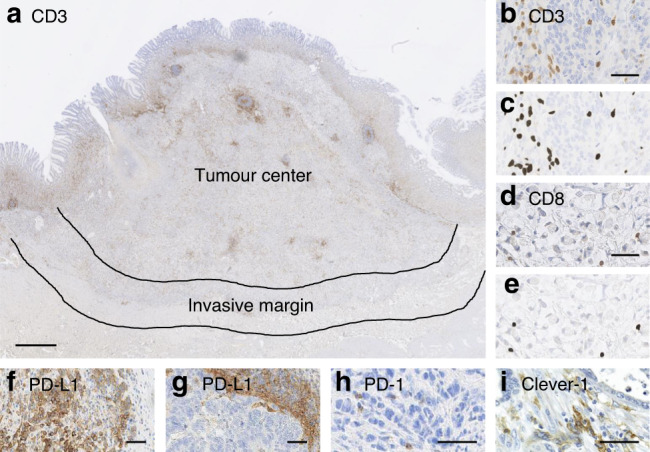
CD3 and CD8 T cell, PD-L1, PD-1 and Clever-1 infiltration in different regions of gastric adenocarcinoma. **a** Image of anti-CD3 stained tissue representing centre of the tumour and the invasive margin used in determination of immune cell score. **b**, **d** Shows CD3 and CD8 positive T cells and scattered tumour cells. (**c**) and (**e**) the corresponding image analysis shows the counted cells (dark grey). **f** PD-L1 positive tumour and **g** positive inflammatory cells. **h** PD-1 positive lymphocytes. **i** Clever-1 positive macrophages. Scale bars are 1 mm (**a**) or 50 µm (**b–i**).

CD3+ and CD8+ immunohistochemistry was assessed by digital image analysis, using ImageJ software and a previously validated cell counting method.^26^ CD3+ and CD8+ immune cell densities were measured from the tumour centre and the invasive margin (Fig. 1), defined as a 0.5-mm wide region on each side of the tumour border. The measurement was also done separately from areas representing the whole region average immune cell infiltrate and from hotspot areas (0.36 mm^2^), defined digitally at the tumour centre and invasive margin, simulating the original tissue microarray (TMA) immune cell score (ICS) protocol. The selected areas for the hot spot analysis were from high cell density areas.

To calculate ICS based on dichotomised CD3 and CD8 densities on the invasive margin and at the tumour centre, receiver operating characteristic (ROC) curves were drawn in relation to 3-year mortality to determine the cut-off values with optimal sensitivity and specificity. The cut-off values for the hotspot densities (cells/mm^2^) in the whole cohort were 1220 for CD3+ at the tumour core, 710 for CD3+ on the invasive margin, 3340 for CD8+ at the tumour core and 1630 for CD8+ on the invasive margin. The cut-off values for the whole section densities were 563, 393, 299 and 361 respectively. In the GC intestinal subgroup, the cut-off values for the hotspot counts were 1550 for CD3+ at the tumour core, 1040 for CD3+ on the invasive margin, 1800 for CD8+ at the tumour core and 1180 for CD8+ on the invasive margin and for the whole section densities were 580, 361, 283 and 425 respectively. In the GC diffuse subgroup, the cut-off values for the hotspot densities were 2410 for CD3+ at the tumour core, 1870 for CD3+ on the invasive margin, 2240 for CD8+ at the tumour core and 750 for CD8+ on the invasive margin and for the whole section densities were 597, 1180, 394 and 291, respectively. Finally, the sum of the dichotomised density variables was calculated, and, according to ICS protocol, three groups were formed: low ICS 0, moderate ICS 1–2 and high ICS 3–4.

For PD-L1 analysis Combined Positive Score (CPS, Fig. 1), which is the number of PD-L1 staining cells (tumour cells, lymphocytes, macrophages) divided by the total number of viable tumour cells, multiplied by 100, was used. The samples were defined to be PD-L1 negative or positive if the PD-L1 CPS was ≤1% or >1%, respectively. The number of PD-1-positive tumour infiltrating lymphocytes (cells/mm^2^) (Fig. 1) was calculated using QuPath. The samples were divided into two groups (PD-1 low and PD-1 high) according to the median value for PD-1 positive lymphocytes (33.30 positive cells/mm^2^). Clever-1 was assessed in three hotspots of cancerous tissue (Fig. 1) with ×40 objective magnification (0.36 mm^2^). Digital images were processed by Pannoramic Viewer version 1.15.4 (3DHISTECH, Hungary). Clever-1 was defined to be high when median ≥15 in macrophages. EBV ISH was scored as either negative or positive according to the nuclear reaction.

### Statistical analysis

Survival times were calculated from the date of surgery until the time of death or the end of follow-up (5 September 2019). The Kaplan-Meier method with log-rank test was used to calculate overall survival (OS) according to the immune cell variables. The relationships between ICS groups and clinico-pathological variables were evaluated by Chi-square test. Univariate and multivariate Cox proportional hazards regression models were used to calculate hazard ratios for survival with the following pre-determined confounders: year of surgery (before 2010, 2010–present), age (<65 years, 65–75 years, >75 years), sex (male, female), tumour stage (I–II and III–IV), Lauren classification (diffuse, intestinal, mixed), adjuvant therapy (yes/no) and radical resection (R0, R1). *P*-value < 0.05 was considered significant. The statistical analyses were performed with IBM SPSS statistics 24 for Windows (IBM Corporation, Armonk, NY, USA).

### Patient demographics

A total of 132 gastric cancer specimens were included in this study. After exclusion of EBV-positive (*n* = 4) and MSI (*n* = 6) tumours, final cohort consisted of 122 patients. The mean age of the cohort was 67.1 years (SD 11.3). TNM stage distribution in our cohort was stage I 27.9% (*n* = 34), stage II 42.6% (*n* = 52), stage III 26.2% (*n* = 32) and stage IV 3.3% (*n* = 4). The detailed data on clinical characteristics and association with ICS according to Lauren Classification are shown in Table 1. Four patients had mixed histology and were not included in the histology-based analyses. Eight patients got perioperative chemotherapy and 54 patients got postoperative adjuvant therapy.

## Results

Median follow-up time of the patients was 37.6 months (IQR 15.0–112.6) and estimated median overall survival 37.5 [95% CI: (15.1–59.8)] months. The 5-year overall survival rate was 43.4%. In the subgroup analysis, overall survival rates were 50.0% in the intestinal subgroup and 35.5% in the diffuse subgroup, respectively.

### Immune cell score

**Fig. 2:**
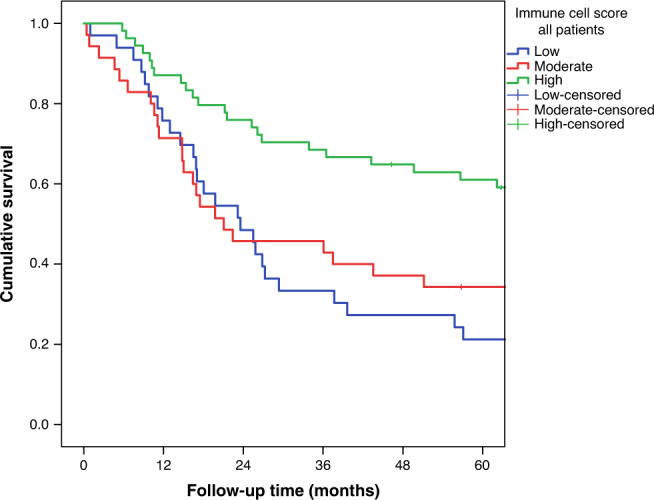
Five-year survival in gastric adenocarcinoma patients stratified by immune cell score. High ICS was associated with improved survival (*p* = 0.001).

Five-year survival in the whole cohort stratified by immune cell score is shown in Fig. 2. Survival curves according to tumour histology are presented in Supplementary Fig. 1a, b.

Hazard ratios (unadjusted and adjusted for confounding factors) of 5-year overall mortality in GC patients with the low (0–1), moderate (2) and high (3–4) immune reactions are shown in Table 2.

**Table 2:** Hazard ratios (HRs) with 95% confidence intervals (CI) of 5-year overall mortality in GC patients with low (0), moderate (1–2) and high (3–4) immune reaction based on ICS.

	Number of patients	Immune cell score 0 HR (95% CI)	Immune cell score 1–2 HR (95% CI)	Immune cell score 3–4 HR (95% CI)
Overall mortality				
All patients (crude)	122	1.00 (Reference)	0.83 (0.47–1.45)	0.36 (0.20–0.64)
All patients (adjusted)^a^	122	1.00 (Reference)	1.10 (0.59–2.03)	0.48 (0.26–0.87)
Subgroup analysis				
Diffuse type (crude)	70	1.00 (Reference)	0.50 (0.26–0.98)	0.25 (0.11–0.55)
Diffuse type (adjusted)^b^	70	1.00 (Reference)	0.93 (0.44–1.97)	0.57 (0.24–1.37)
Intestinal type (crude)	48	1.00 (Reference)	0.82 (0.31–2.18)	0.43 (0.16–1.15)
Intestinal type (adjusted)^b^	48	1.00 (Reference)	1.06 (0.33–3.43)	0.39 (0.12–1.25)

^a^Adjusted for year of surgery (before 2010, 2010–present), age (<65 years, 65–75 years, >75 years), sex, tumour stage (I–II and III–IV), Lauren Classification (diffuse, intestinal, mixed), adjuvant therapy (yes/no) and radical resection (R0, R1).

^b^Adjusted for year of surgery (before 2010, 2010–present), age (<65 years, 65–75 years, >75 years), sex, tumour stage (I–II and III–IV), adjuvant therapy (yes/no) and radical resection (R0, R1).

### Immune cell score in whole section and hotspot analysis

In whole section analyses the respective 5-year overall survival rates for the low, moderate and high ICS groups were 21.2%, 34.3% and 61.0%, *p* = 0.001. In the diffuse subgroup there was statistical significance in the 5-year overall survival rate for low, moderate and high ICS groups 8.7%, 37.5% and 60.9%, *p* = 0.001. In hotspots, there was no statistically significant difference between low, moderate or high ICS in the entire cohort or in the subgroups. Results in the entire cohort and subgroup analyses are shown in Table 3.

**Table 3:** Median OS (months) and 5-year survival rates according to low, moderate and high ICS groups.

	ICS low (0)	ICS moderate (1–2)	ICS high (3–4)	
Whole section				
All	23.6 (95% CI 14.8–32.3) 21.2%	21.1 (95% CI 0.00–43.3) 34.3%	86.3 (95% CI 49.7–122.9) 61.0%	*p* = 0.001
Intestinal	25.8 (95% CI 23.5–28.1) 33.3%	37.5 (95% CI 23.8–51.2) 42.9%	67.4 (95% CI 47.3–87.5) 63.6%	*p* = 0.219
Diffuse	16.9 (95% CI 16.1–17.7) 8.7%	22.4 (95% CI 0.00–61.0) 37.5%	171.4 (95% CI 32.2–310.6) 60.9%	*p* = 0.001
Hotspot				
All	23.2 (95% CI 2.0–44.3) 10.0%	43.5 (95% CI 21.2–65.9) 44.3%	56.6 (95% CI 10.8–102.5) 48.3%	*p* = 0.104
Intestinal	26.8 (95% CI 23.4–30.3) 14.3%	79.6 (95% CI 15.4–143.8) 56.5%	62.1 (95% CI 46.8–77.4) 55.6%	*p* = 0.062
Diffuse	23.2 (95% CI 10.8–35.5) 20.0%	22.4 (95% CI 0.00–48.3) 37.5%	21.5 (95% CI 0.00–170.1) 46.7%	*p* = 0.732

### PD-L1, PD-1 and Clever-1

We observed PD-L1 positive CPS (>1%) in 46 (37.7%) patients, of whom 28 (60.9%) had high ICS. The corresponding numbers regarding high PD-1 positive lymphocyte density were 57 (46.7%) patients, of whom 36 (63.2%) had high ICS and regarding high Clever-1 positive macrophage density 50 (41.0%) patients, of whom 24 (48.0%) had high ICS.

In the whole cohort, PD-L1 CPS (*p* = 0.474) was not statistically associated, and no trends were observed with survival and similar results were seen between low and high densities of PD-1 positive lymphocytes (*p* = 0.204) and Clever-1 positive macrophages (*p* = 0.428).

**Fig. 3:**
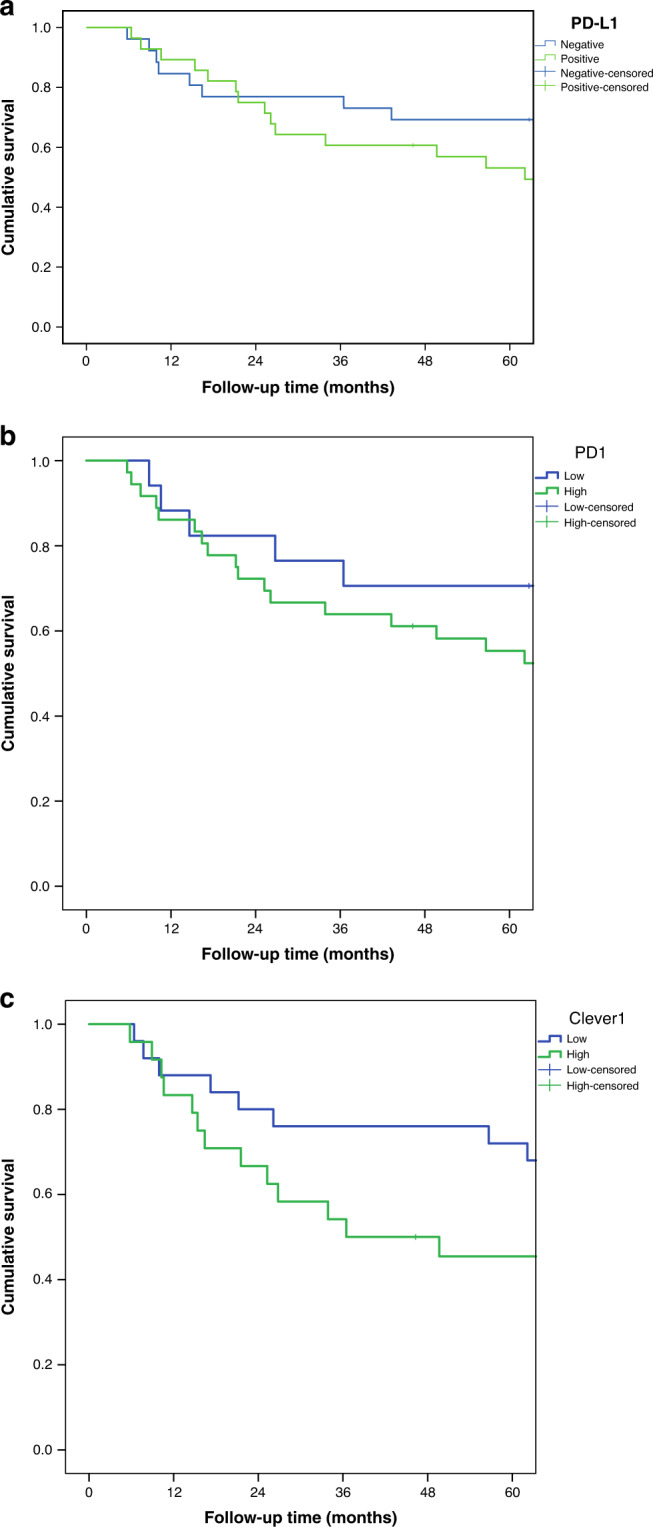
In the high ICS group, patients with high PD-L1, PD-1 and Clever-1 had poor prognosis. **a–c** Effect of PD-L1 expression (**a**), low and high PD-1 (**b**) and Clever-1 (**c**) expression on patient survival. Only high ICS tumours are included.

When including only tumours with high ICS, the 5-year survival in cases with CPS negative vs. positive PD-L1 was 69.2% vs. 53.1% (*p* = 0.317, Fig. 3a); for PD-1 positive lymphocytes 70.6% vs. 55.3% (*p* = 0.312, Fig. 3b); and for Clever-1 positive macrophages 72.0% vs. 45.5% (*p* = 0.070, Fig. 3c). Hazard ratios (unadjusted and adjusted) are presented in Table 4.

**Table 4:** Hazard ratios (HRs) with 95% confidence intervals (CI) of the 5-year overall mortality in GC patients with high immune cell score stratified by PD-L1, PD-1 and Clever-1.

	Number of patients	PD-L1 CPS negative, HR (95 % CI)	PD-L1 CPS positive, HR (95 % CI)
5-year overall mortality			
High ICS patients (crude)	54	1.00 (Reference)	1.56 (0.65–3.78)
High ICS patients (adjusted)^a^	54	1.00 (Reference)	1.20 (0.46–3.13)
		PD-1 Low, HR (95 % CI)	PD-1 High, HR (95 % CI)
High ICS patients (crude)	54	1.00 (Reference)	1.65 (0.61–4.51)
High ICS patients (adjusted)^a^	54	1.00 (Reference)	1.88 (0.53–6.75)
		Clever-1 Low, HR (95 % CI)	Clever-1 High, HR (95 % CI)
High ICS patients (crude)	54	1.00 (Reference)	2.27 (0.90–5.71)
High ICS patients (adjusted)^a^	54	1.00 (Reference)	2.24 (0.69–7.29)

^a^Adjusted for year of surgery (before 2010, 2010–present), age (<65 years, 65–75 years, >75 years), sex, tumour stage (I–II and III–IV), Lauren Classification (diffuse, intestinal, mixed), adjuvant therapy (yes/no) and radical resection (R0, R1).

### Microsatellite instability and Epstein-Barr virus status

The original cohort included six MSI tumours and four EBV-positive (EBV+) tumours, which were excluded from the analyses. All these patients had intestinal GC. Mean age of MSI patients was 74 years and three patients were men. ICS status was high in four patients, moderate in one and low in one patient. High PD-1 was seen in all patients and high Clever-1 in three patients. In tumour cells PD-L1 was high in three patients and in inflammatory cells in four patients. Three patients died during 12-month follow-up and one patient during 36-month follow-up. Mean age of EBV+ patients was 77.8 years, and two patients were men. All these patients had intestinal GC. ICS, PD-1 and Clever-1 -status was high in all patients. In tumour cells PD-L1 was high in three patients and in inflammatory cells in all patients. Two patients died during 12-month follow-up and one patient during 36-month follow-up.

## Discussion

The main finding of this study indicates that high ICS is associated with improved overall survival in GC patients. Furthermore, in the high ICS group patients with high PD-L1, PD-1 or Clever-1 had poor prognosis, highlighting the role of immune escape and immune tolerance in GC. Whole section analysis seems to be a more accurate and reliable method than hotspot analysis.

Significant differences between high and low ICS (combination of CD3, CD8, CD45 and CD66 from the invasive margin and centre of the tumour) patients were previously seen in both 5-year disease-free survival and overall survival of GC patients (*n* = 125).^9^ Furthermore, ICS was shown to be an independent prognostic factor and a combination of ICS and TNM stage had better prognostic value than TNM stage alone.^9^ The study did not report results according to histological subtype.^9^ Diffuse and intestinal GCs differ in molecular tumorigenesis and clinical and histopathological features.^27^ Intestinal GC is related to chronic Helicobacter pylori infection resulting in atrophic gastritis with intestinal metaplasia. Chronic inflammation causes a cascade atrophic gastritis and intestinal metaplasia that may be a precursor of cancer or alternately only a marker of long-term gastric atrophy.^27^ Our results suggest that ICS may be more accurate in diffuse GC. We hypothesise that, the pre-existing inflammation in intestinal tumours may confound the interpretation of the host immune response, resulting in ICS is being more useful in the diffuse subtype.

Approximately 7–9% of GC patients have a high level of MSI according to findings in recent meta-analysis.^28,29^ MSI is usually associated with intestinal type GC^28^ and this was also seen in our study. Previously, 33% and 45% PD-L1+ staining was seen on tumour cells and immune cells in MSI high tumours.^30^ Based on molecular classification of GC, ~4–9% of GC patients are EBV+ tumours.^4,31^ Previously PD-L1 positivity in tumour and inflammatory cells was reported in 77% and 100% of EBV+ tumours.^31^ In our study out of four EBV+ tumours, respective numbers were 75% and 100%. According to Lauren Classification, PD-L1+ in tumour cells seem to be slightly more common in intestinal type, although without major differences.^31^ Effect on prognosis has not been reported.

Our results suggest that the combined evaluation of ICS and PD-L1 is associated with overall survival in GC, and this immune classification could represent a potential addition in GC staging with TNM. So far only a few studies have been presented on ICS combined with PD-L1 and their association with prognosis of GC. Wang et al. recently classified their patients into two subgroups according to strong and weak immunoreaction defined by the number of CD8+ T cells and PD-L1 expression in tumours. Patients with weak immune reaction lived shorter time than patients with strong immune reaction displaying high CD8+ cells and low PD-L1 expression.^18^ In another study, in multivariate analysis combined status of PD-L1 and ICS was an independent and significant prognostic factor for overall survival of GC patients in MSI-high GC patients.^32^ In a recent study genetic deficiency of Clever-1 impaired solid tumour growth, activated endogenous antitumour CD8+ cells and these effects were similar to PD-1 checkpoint inhibition, thereby supporting the notion that combining anti-Clever-1 with anti-PD-1 provides synergistic benefit in aggressive, nonresponsive tumours.^21^

Host immunity has a critical role in controlling cancer development and progression. Some GCs are associated with a rich immune infiltrate which may have a positive impact on responsivity to immunotherapies. In GC patients the clinical benefit and improved survival when treated with immunotherapeutic strategies and combined with conventional therapies highlights the importance of the immune environment surrounding the tumour.^12^ Earlier studies have reported that PD-L1 is expressed in ~65% of GC.^12^ Furthermore, PD-L1 expression in tumour cells has been reported in 14–24% of patients and in immune cells in ~35% of patients with gastro-oesophageal cancer.^16^ Tumours with a rich immune infiltrate are considered more responsive to checkpoint inhibitors.^15,30^ There are several ongoing studies in Phase 1–3 with various combinations and different settings against PD-L1 positive GC (metastatic, unresectable or recurrent disease or advanced tumours).^12,15^ In a study on 259 recurrent or metastatic GC patients treated with Pembrolizumab (57.1% PD-L1 positive) objective response rate (ORR) was 15.5% among PD-L1-positive tumours compared to 6.4% in PD-L1-negative tumours.^33^ Furthermore, seven of the evaluable tumours were MSI-H and ORR was 57.1% compared to non-MSI-H tumours 9.0%.^33^ However, it is partly unclear which subgroups should be treated and what combinations of immunotherapy should be used. Our study provides new information on the distribution of PD-L1 and PD-1 expression in different histological subtypes of GC and according to the extent of T cell infiltrate. According to our study and the findings of earlier studies, checkpoint inhibitors may be especially useful in high ICS, PD-L1 positive tumours.

High ICS in whole sections in our study predicted survival more accurately than hotspot analyses. Hotspots simulating TMA technique showed a similar trend without statistical significance. Few studies comparing hotspot and whole section analysis to count immune cell infiltrates have been presented and as far as we know none of these were performed on GC samples.^11^ The problem with hotspots is that this region represents only a small part of the whole tumour area and immune cell densities change from one level of the section to the next.

The strength of this study is a consecutive series of D2 total or subtotal gastrectomy patients from 1997 to 2016 from a single geographical area of Central Finland without apparent selection bias. Prospective data collection and double checking of the hospital records by another researcher not responsible for treating these patients was performed. All patients were followed up in Central Finland Central Hospital for up to 5 years after surgery and nationwide compulsory databases enabled us to obtain complete long-term mortality data. A major limitation of the study is sample size, limiting the subgroup analyses, where possible positive associations may be missed due to low statistical power. Confidence intervals for reported hazard ratios are wide and replication studies are needed to confirm the findings. The status of Helicobacter pylori was not determined in the study population. There were only six patients with MSI and four patients with EBV+ status thus rendering sensible subgroup analyses unfeasible.

## Conclusions

High ICS was associated with improved overall survival in GC. In the high ICS group, patients with high PD-L1, PD-1 or Clever-1 had poor prognoses, highlighting the importance of immune tolerance in GC.

## Supplementary information

Supplementary data

## Data Availability

Anonymised data is available upon request from the corresponding author.
