# Supplementary material for: Immunophenotype based on inflammatory cells, PD-1/PD-L1 signalling pathway and M2 macrophages predicts survival in gastric cancer
Source: Br J Cancer. 2020 Sep 18;123(11):1625–32. doi: 10.1038/s41416-020-01053-7 (PMC7687887; doi:10.1038/s41416-020-01053-7)

**Supplementary data for article:**

**”Immunophenotype Based on Inflammatory Cells, PD-1/PD-L1 Signalling Pathway and M2 Macrophages Predicts Survival in Gastric Cancer”**

Junttila Anna^1*^, Helminen Olli^12^, Väyrynen Juha P^2,3,4^, Ahtiainen Maarit^5^, Kenessey Istvan^6^, Jalkanen Sirpa^6^, Mecklin Jukka-Pekka^7^, Kellokumpu Ilmo^1^, Kuopio Teijo^5,8,9^, Böhm Jan^8^, Mrena Johanna^1^

^1^ Department of Surgery, Central Finland Central Hospital, Jyväskylä, Finland

^2^ Cancer and Translational Medicine Research Unit, Medical Research Center Oulu, University of Oulu, and Oulu University Hospital, Oulu, Finland

^3^ Department of Medical Oncology, Dana-Farber Cancer Institute and Harvard Medical School, Boston, MA, USA

^4^ Department of Pathology, Brigham and Women’s Hospital, Boston, MA, USA

^5^ Department of Education and Research, Central Finland Health Care District, Jyväskylä, Finland

^6^ MediCity Research Laboratory and Institute of Biomedicine, University of Turku, Turku, Finland

^7^ Department of Education and Research, Central Finland Central Hospital and Sport and Health Sciences, University of Jyväskylä, Jyväskylä, Finland

^8^ Department of Pathology, Central Finland Central Hospital, Jyväskylä, Finland

^9^ Department of Biological and Environmental Science, University of Jyväskylä, Jyväskylä, Finland

***Corresponding author:**

Anna Junttila

Department of Surgery, Central Hospital of Central Finland, Jyväskylä, Finland

Keskussairaalantie 19

40620, Jyväskylä, Finland

Tel: +358443300274

E-mail: [anna.junttila@fimnet.fi](mailto:anna.junttila@fimnet.fi)

**Figure legends:**

**Supplementary Figures 1a and b.**  Five-year survival in intestinal (a) and diffuse (b) gastric adenocarcinoma patients stratified by immune cell score.


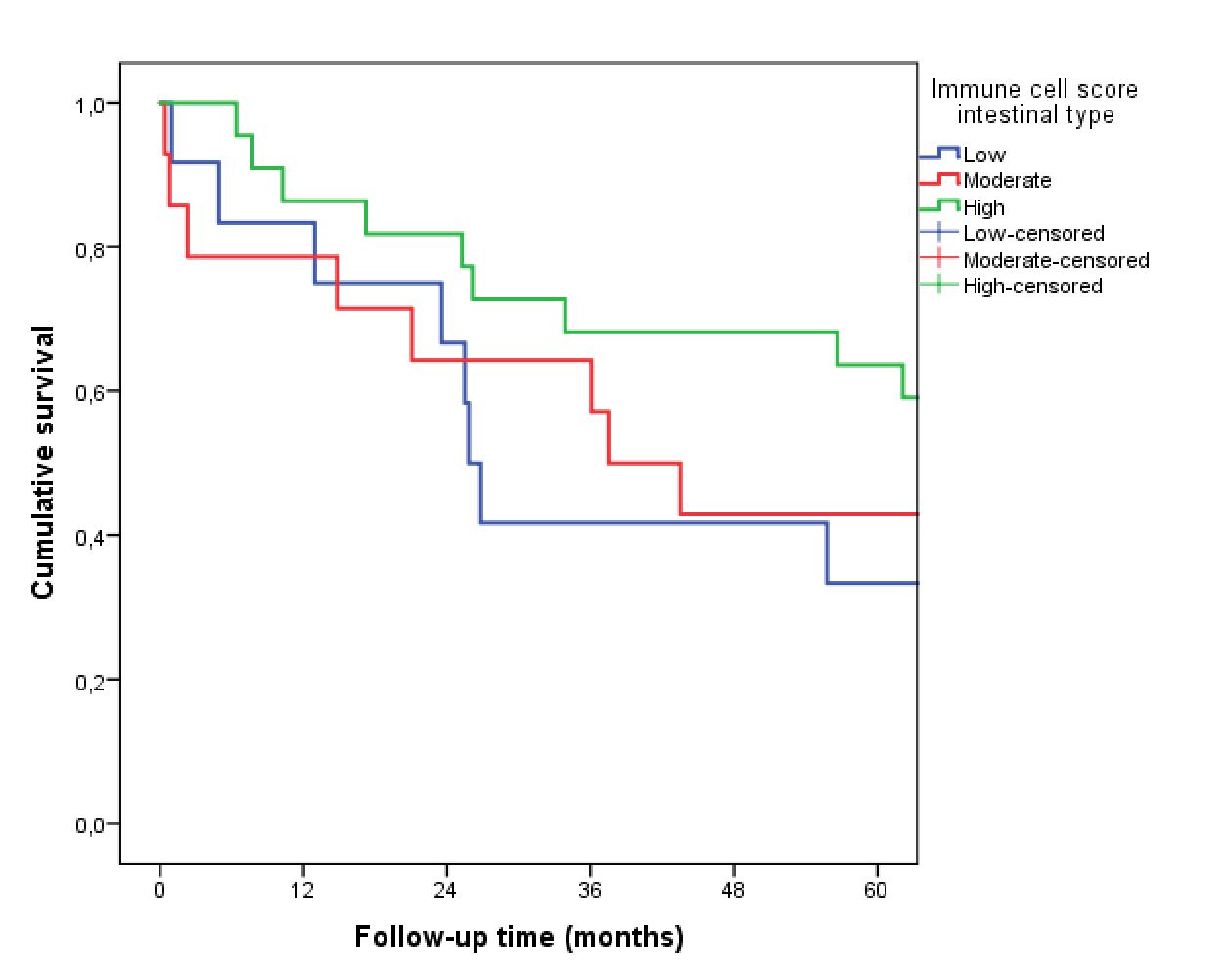


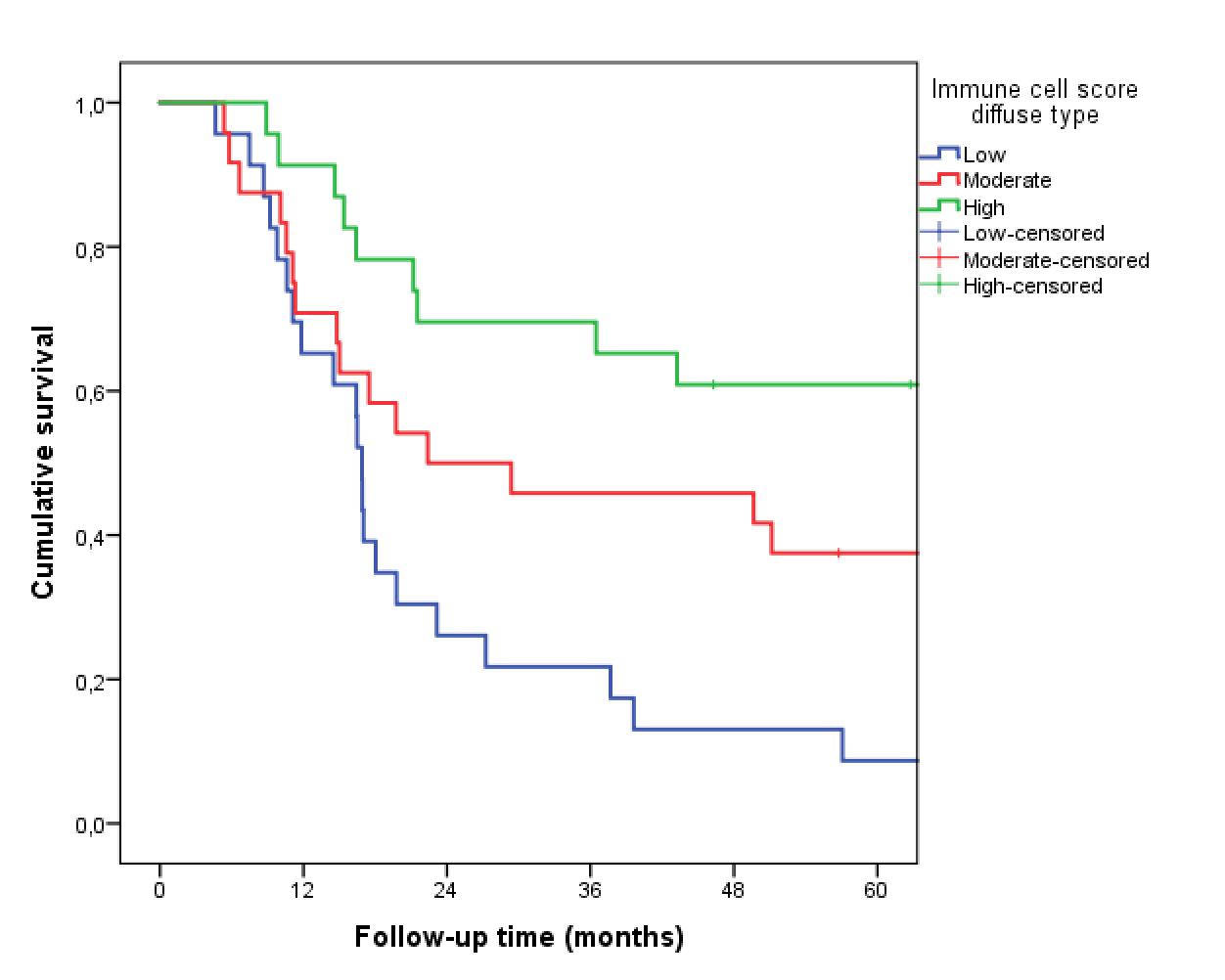

Supplement: Supplementary file 1 — Supplementary data [file 41416_2020_1053_MOESM1_ESM.docx]
